# Intramembranous bone formation after callus distraction is augmented by increasing axial compressive strain

**DOI:** 10.1371/journal.pone.0195466

**Published:** 2018-04-06

**Authors:** Julian Schuelke, Nicholaus Meyers, Sandra Reitmaier, Svenja Klose, Anita Ignatius, Lutz Claes

**Affiliations:** Institute of Orthopedic Research and Biomechanics, Center of Musculoskeletal Research Ulm, University Hospital Ulm, Ulm, Baden-Württemberg, Germany; University of Zaragoza, SPAIN

## Abstract

The mechanical environment is a primary factor in the success of distraction osteogenesis. It is known that the interfragmentary movement during the distraction and maturation phase effects the callus formation. In addition to cyclic compression, other movements like shear and bending influence the bone formation process as shown in previous callus distraction studies. Reports of cartilage presence and endochondral ossification in the regenerative zone have been associated with a lack of fixation stability and delayed healing. So far the effects of the direction of interfragmentary movements could not be studied separately. By means of a unique lateral callus distraction model, we investigated the effects of small (0.1 mm) and moderate (0.6 mm), purely axial compression on ossification during callus maturation in sheep. A distraction device incorporating a mobile titanium plate was mounted on the tibia. Following lateral callus distraction, electromechanically controlled movements allowed purely axial cyclic compression of the tissue regenerate. Seven weeks post-operatively, the tissue regenerates were investigated using μCT, histology and immunohistochemistry. The larger amplitude significantly increased bone formation (Fractional bone volume: 19.4% vs. 5.2%, *p* = 0.03; trabecular thickness: 0.1 mm vs. 0.06 mm, *p* = 0.006; mean spicule height: 2.6 mm vs. 1.1 mm, *p* = 0.02) however, no endochondral ossification occurred. The elimination of shear movement, unimpaired neovascularization as well as the tensile strain stimuli during the distraction phase suppressing chondrogenic differentiation may all contribute to the absence of cartilage. In clinical application of distraction osteogenesis, moderate axial interfragmentary movement augments intramembranous ossification provided shear strain is minimized.

## Introduction

Distraction osteogenesis (DO) is a commonly used method for the correction of skeletal defects. The so called “tension-stress effect” utilizes mechanical tension to induce bone formation [1]. However, DO is susceptible to delayed healing and other complications associated with the protracted use of external fixation such as pin tract infection and screw loosening [2]. In addition to the distraction stimulus, Ilizarov reported that the extent of osteogenic activity within a distraction zone depends upon the stability of the external fixator construct [1]. Reports of cartilage presence and endochondral ossification in the regenerative zone have been associated with a lack of fixation stability or distraction patterns exceeding physiological tolerance in time or magnitude, respectively, and lead to delayed healing [1, 3]. The 3-dimensional fixator stiffness and the physiological loading due to weight bearing and muscle forces determine the 3-dimensional deformation in the fracture gap. In addition to the predominant compression other movements like shear and bending occur and their influence on the bone healing process are not known. Loading and dynamization with moderate, predominantly axial compression after DO improved bone formation compared to either no or large predominantly axial compression [4–6]. The effect of different pure cyclic compression to the bone healing after callus distraction however is not known.

Previously, Claes et al. implemented a model of lateral callus distraction for bone formation without the conventional splitting and weakening of the bone [7]. A distraction device mounted anteromedially on the intact ovine tibia allowed distraction of newly-formed granulation tissue. The tissue developed between a flat periosteum-resected area of the bone and the porous, hydroxyapatite-coated titanium plate, anchored by ingrowth. By distracting the titanium plate laterally 0.275 mm twice daily for ten days, callus formation was stimulated between the bone surface and the titanium plate. During the following maturation period, deformation of the regenerating tissue under ambulatory loading was prevented by the stiff fixator configuration connected to the intact tibia. Under these conditions, intramembranous ossification of the newly formed callus was observed. To determine the effects of pure axial compression magnitude on ossification after lateral callus distraction, a modified custom-made distraction device was used to apply two different compression amplitudes (0.1 mm and 0.6 mm) on the regenerating tissue by cyclic movement of the plate. The aim of this study was to investigate the effects of pure cyclic compression on bone formation and tissue differentiation after DO while avoiding the drawbacks of osteotomy models, e.g. multi-modal deformation.

## Materials and methods

### Study design

**Fig 1:**
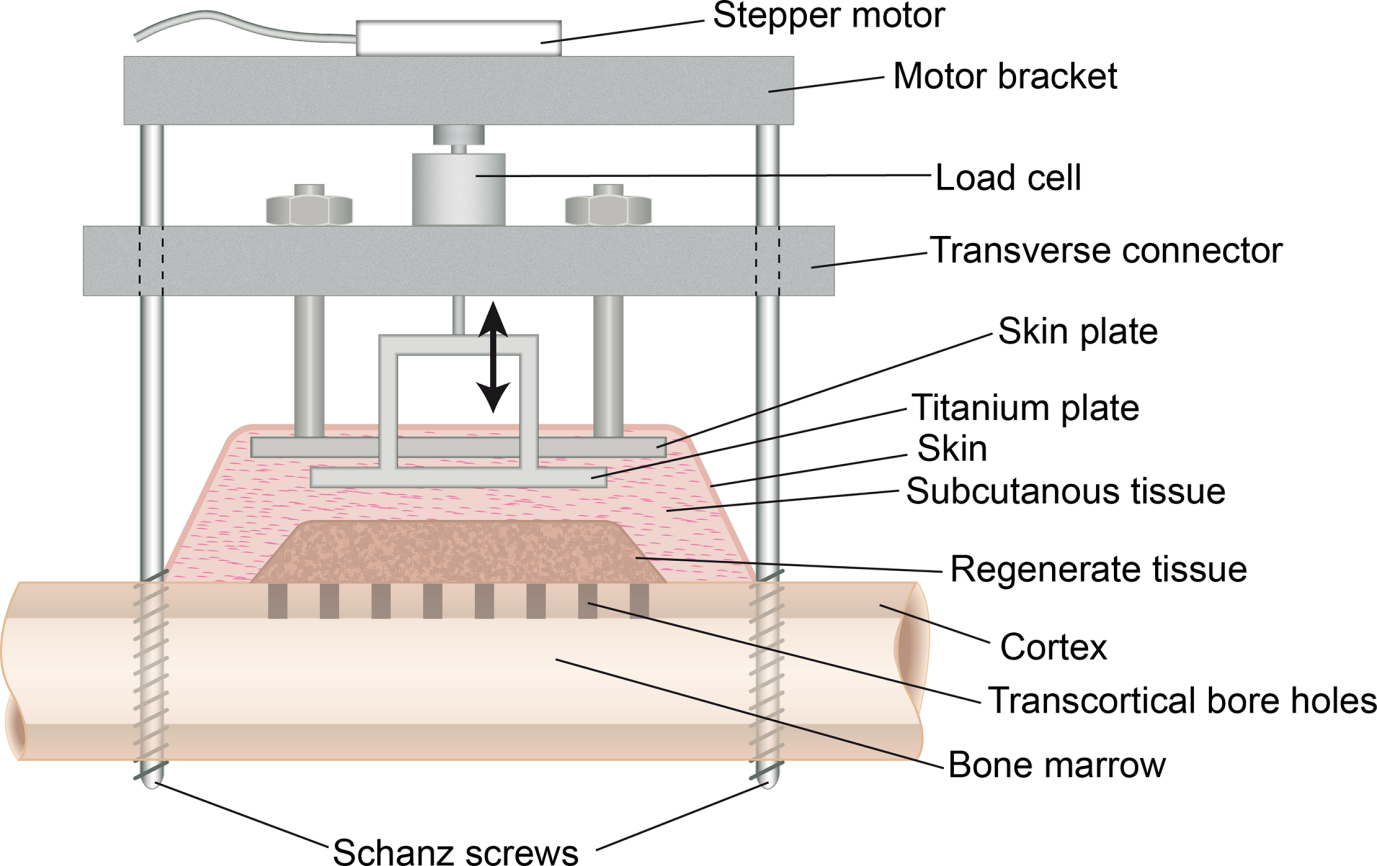
Distraction device. The distraction device consisted of two Schanz screws in combination with the transverse connector and created a rigid frame. To minimize interference with our measurements, the skin plate elevated the skin above the titanium plate.

**Fig 2:**
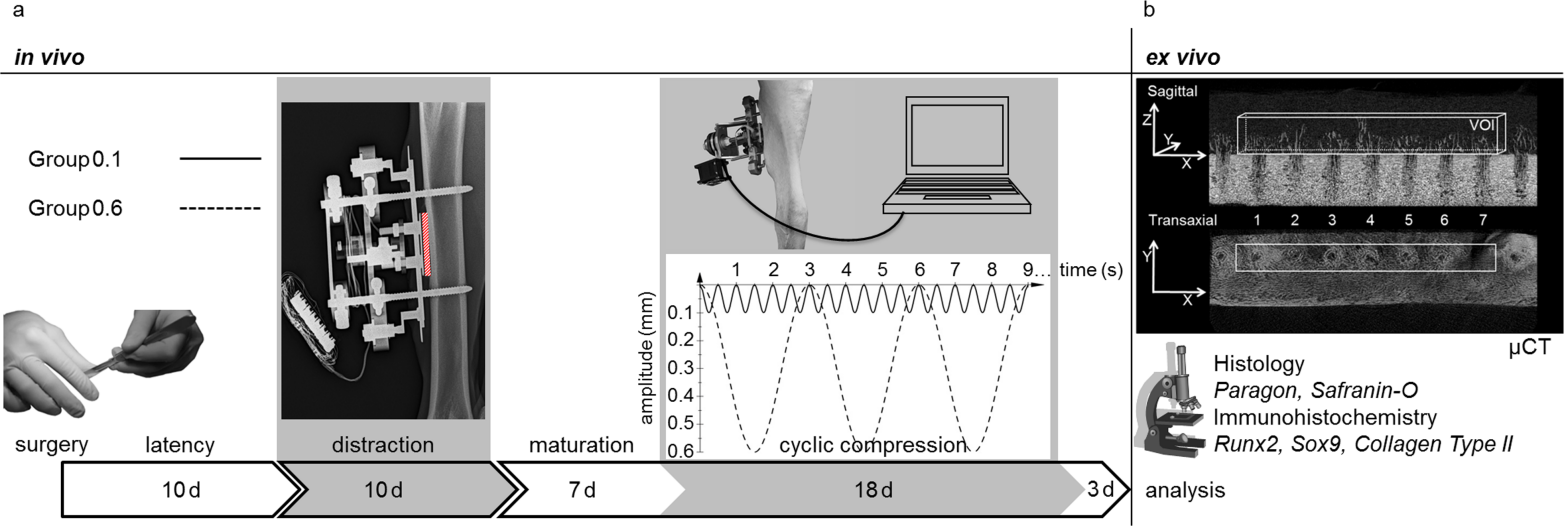
**a-b. Experimental set up *in vivo* and *ex vivo*.** The *in vivo* experiment (Fig 2A) consisted of two phases of manipulations (grey areas). Following surgery and latency phase, the animals were subjected to ten days of lateral callus distraction creating a gap of regenerating tissue between tibial cortex and titanium plate (hatched area in X-ray). Seven days into maturation, the regenerating tissue received electromechanically induced cyclic compression of two differing amplitudes and frequencies (Group 0.1 is depicted by the black line; Group 0.6 is depicted by the dotted line). The *ex vivo* analysis was performed by μCT, histology and immunohistochemistry (Fig 2B). Volume of interest (VOI) dimensions for μCT analysis are shown using exemplary sagittal and transaxial images.

In an animal experiment employing sheep, a distraction device (Fig 1) incorporating a mobile hydroxyapatite-coated titanium plate (CelGen AG, Zug, Switzerland) was mounted to the right tibia [8]. Postoperatively, the animals were subjected to a combined protocol of lateral callus distraction and subsequent cyclic, compressive stimulation of the regenerate tissue through movement of the titanium plate (Fig 2A). Seven weeks postoperatively, the animals were euthanized by penetrating captive bolt and exsanguination. The specimens were analyzed for quantity of bone formation and mode of ossification using μCT and histology as well as immunohistochemistry (Fig 2B).

The distraction device allowed the distraction and cyclic movement of the titanium plate, avoiding uncontrolled motion due to muscle forces, skin forces, or external forces (load bearing). The stepper motor was attached to the system only during the distraction and cyclic compression using a motor bracket. A load cell integrated in the distraction device allowed the measurement of the forces acting during distraction as well as during cyclic compression. This provided a means of monitoring the open loop control and generating data for another study. The transcortical bore holes allowed the vascularization of the newly regenerated tissue from the bone marrow.

All interventions on living animals were performed according to the regulations of EU Directive 2010/63/EU for animal experiments and conformed to ARRIVE guidelines (Animal Research: Reporting of in Vivo Experiments). The experiments were approved by the local ethics committee (Registration.-no. 1168, Regierungspräsidium Tübingen, Germany). For the animal experiment, 12 adult female Merino sheep (age: 4–6 years; weight: 75–100 kilograms) were operated in two series of six and at time of surgery, randomly distributed in two groups of differing stimulation amplitudes (0.1 mm and 0.6 mm).

In this study, n = 5 (Group 0.1) and n = 6 (Group 0.6) were analyzed. For histologic analysis, the number of specimens investigated was reduced to n = 4 (Group 0.1) and n = 5 (Group 0.6) due to severe staining artifacts in two specimens.

### Surgery

Surgery and perioperative medical treatment followed the protocol described by Claes et al [7]. In brief, the skin at the medial aspect of the right tibia was incised and prepared to visualize the bone surface. Two Schanz screws (DePuy Synthes Companies, Zuchwil, Switzerland), Ø 5 mm proximally and Ø 4 mm distally, were inserted in a standardized position perpendicular to the long bone axis and 55 mm apart. After resection of the periosteum in a defined rectangular field between the Schanz-screws, a flat plane was milled into the cortex, approximately 0.5 mm in depth. The plane was created in accordance with the titanium plate dimensions (35 x 10 mm). Subsequently, 22 holes, 1.1 mm in diameter were drilled in two parallel rows, 3 mm apart, into the medullary cavity underneath the prepared site to allow sufficient neovascularization of the regenerating tissue. The titanium plate was placed on the cortical surface and enclosed following skin adaptation. Ultimately, the distraction device was mounted to the Schanz screws with the titanium plate held in position by a threaded connection to a load cell and shaft coupling in series.

### Post-operative protocol

Post-surgery, the animals were housed in pairs on straw bedding with water and hay provided *ad libitum*. General health was assessed daily with regular cleaning of the surgical wound. The experiment duration was 49 days, divided into ten days post-surgical latency, ten days distraction, seven days maturation without manipulations, then eighteen days cyclic compression and a three day rest (Fig 2A). During lateral callus distraction the titanium plate was elevated by 0.275 mm perpendicular to the long axis of the bone twice daily, resulting in a 5.5 mm gap at the end of distraction phase (Fig 2A). Following the seven-day maturation phase, the regenerate in the gap between tibial cortex and titanium plate was stimulated by cyclic compression using a removable stepper motor/linear actuator (CanStack linear actuator, West Chester, Pennsylvania, USA) once daily. Therefore, the titanium plate fixation was unlocked to allow motor controlled motion. In detail, Group 0.1 was stimulated with 0.1 mm amplitude at a frequency of 2 Hz for one minute, Group 0.6 received 0.6 mm amplitude at 0.33 Hz for six minutes resulting in 120 cycles at a constant initial strain rate in both groups (Fig 2A). The literature indicates that the number of stimulation cycles plays an important role in bone formation [9, 10]. Furthermore, an *in vitro* study indicates that cell proliferation does *not* significantly depend on the frequency of cyclic strain when the cycle number is held constant [9] while an *in vivo* study on sheep tibiae shows that an axial stimulation controlling for magnitude and total number of cycles does *not* show any influence on the healing for frequencies of 1, 5, and 10 Hz [11]. Because the strain rate may have a more pronounced effect on the bone formation than the loading frequency within the range applied in this experiment, we kept the initial strain rate (plate velocity, 0.4 mm/s) between groups of different amplitudes constant and controlled the total number of cycles.

### Preparation

After sacrifice, the right tibia was explanted and the distraction device detached. The tissue regenerate was cut between the two rows of medullary boreholes leaving one slice for radiological and histological analyses and the other for immunohistochemistry.

### μCT

The specimens were scanned without medium at 100 kVp, 100 μA, 2065 ms integration time and a Voxel size of 15 μm^3^ (Skyscan 1172, Skyscan, Kontich, Belgium). After reconstruction of the data set, the volume of interest (VOI) was set to a width of 2.2 mm (two times the bore hole diameter), 21 mm in length (including seven bore holes in the middle of the row) and 3 mm in height starting from the lowest plane without cortical bone fragments (Fig 2B). Analysis was performed using an analysis software (CTAnalyser, Bruker, Billerica, Massachusetts, USA) with the lower grey threshold set at 50 (20% of upper grey threshold) to investigate trabecular thickness (Tb.Th.), trabecular number (Tb.N.), trabecular separation (Tb.Sp.), and fractional bone volume (BV/TV). Furthermore, the height of the conic osseous structures above the medullary bore holes, referred to as spicules, was measured. Using DataViewer (Bruker, Billerica, Massachusetts, USA) the height of each single spicule and the mean height of spicules for each specimen were calculated.

### Histology

Undecalcified histological samples were obtained by embedding formalin fixed specimens in polymethyl methacrylate (PMMA, Roth GmbH, Karlsruhe, Germany). The ground sections (70 μm) were stained with Paragon (Waldeck GmbH, Münster, Germany) and evaluated using light microscopy (Leica DMI6000B, Leica microsystems, Wetzlar, Germany) at each available magnification between 12.5–400 x. The regenerate area was assessed qualitatively regarding tissue types. Tissue parameters investigated were bone including osteoid (B), cartilage (Ca), fibrous tissue (Fb), fat tissue (Fa), and vessels (V). Cellular morphology was studied regarding state of differentiation and metabolic activity. Histomorphometry was performed using point counting analysis. An area in the middle of the regenerate covering 12 mm in width and 3 mm in height was scanned with a grid. The grid fields (12 x 3) with 100 count points each added to 3600 points. The number of points sufficed to present the relative values of tissue parameters investigated with < 5% error [12].

For paraffin embedded slices, specimens were decalcified in EDTA and cut at 5–7 μm. For detection of cartilage typical proteoglycan production, paraffin sections were stained with Safranin-O (Waldeck GmbH, Münster, Germany).

### Immunohistochemistry

Immunohistochemistry was performed on paraffin sections with a monoclonal *anti*-Runx2 antibody (#8486; Cell Signaling Technology corp., Cambridge, UK) for evaluation of osteogenic differentiation and with two polyclonal antibodies, *anti*-Sox9 (NBP2-24659, Novus Biologicals LLC, Littleton, Colorado, USA) and *anti*-Collagen Type II (#600-401-104, Rockland Immunochemicals, Limerick, Pennsylvania, USA) to detect cartilaginous differentiation. Briefly, after routine preparation with xylol and rehydration in decreasing alcohol concentrations, unmasking of antigen epitopes was performed at 85° C in buffered citrate or using *Target Retrieval Solution* (DAKO, Agilent Technologies, Santa Clara, California, USA). 5% goat-serum in TTBS (TBS incl. 0.1% Triton X-100) at 37°C was used for blocking before incubating slices with the primary antibody (1/100) at 4°C overnight. Application of an Avidin-Biotin-complex (Vectastain ABC Kit#PK-6100, Vector Laboratories, Burlingame, California, USA) and substrate (Nova Red, Vector #SK-4800, Vector Laboratories, Burlingame, California, USA) completed preparation.

### Statistics

The bone structural parameter data from μCT analysis (BV/TV, Tb.Th., Tb.N., Tb.Sp.) was examined for normal distribution using the Shapiro-Wilk-test. Differences between groups were identified using the parametric, double t-test with subsequent Bonferroni-Holm adjustment as a post hoc-test. Histomorphometry was compared using the Mann-Whitney-U-test for non-parametric data due to lower specimen numbers (Group 0.1: n = 4 and Group 0.6: n = 5) barring normal distribution. Statistical analysis was performed using GraphPad software (GraphPad Software Inc., La Jolla, California, USA). The level of significance was set at α = 0.05.

## Results

### Surgery and postoperative protocol

Surgeries were performed successfully with animals recovering to full weight bearing after about three days. The manipulations during distraction and cyclic stimulation were well tolerated. The sheep showed no signs of pain or discomfort during manipulations. One sheep was excluded due to immobilization of the titanium plate resulting from excessive serous fluid excretion.

### μCT

**Fig 3:**
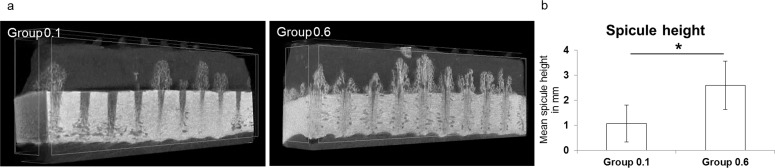
**a-b. Representative** μ**CT images and mean spicule height.** The representative μCT-images show variant new bone formation between experimental groups (Fig 3A). The mean spicule height between groups was significantly different (*p = 0.02, data given as mean with standard deviation; Fig 3B).

**Fig 4:**
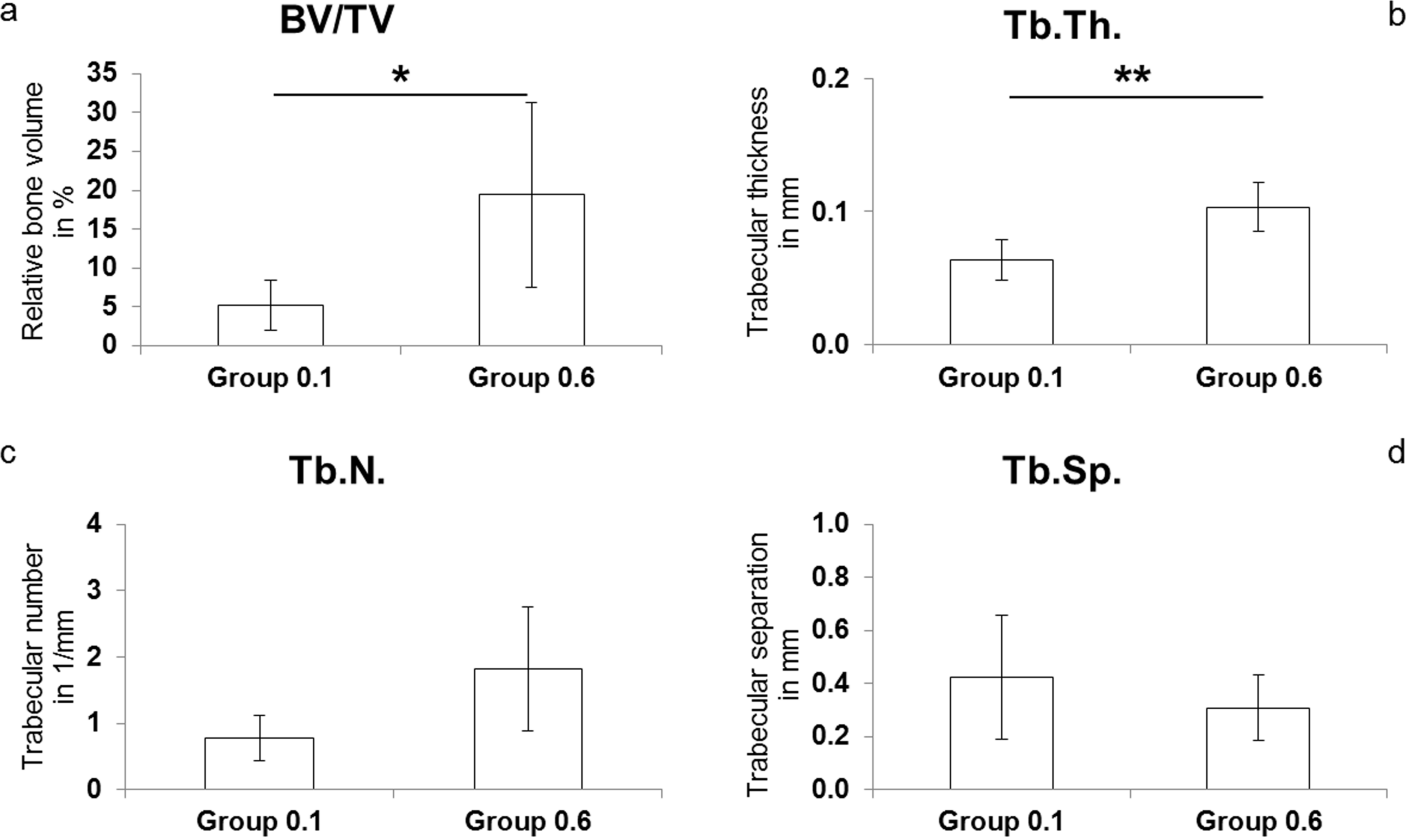
**a-d. Bone structural parameter data.** The bone structural parameter data from μCT analysis shows differences of relative bone volume (BV/TV, *p = 0.03; Fig 4A), trabecular thickness (Tb.Th., **p = 0.006; Fig 4B), trabecular number (Tb.N.; Fig 4C) and trabecular separation (Tb.Sp.; Fig 4D).

μCT analysis revealed significant differences in bone formation between the groups (Figs 3 and 4A–4D). The mean bone spicule height reached significantly different levels with 2.6 mm (SD ± 1.0) in Group 0.6 compared to 1.1 mm (SD ± 0.7) in Group 0.1 (*p* = 0.02; Fig 3B). BV/TV was approximately four-fold higher in Group 0.6 (19.4%; SD ± 11.8) compared to Group 0.1 (5.2%; SD ± 3.2; *p* = 0.03; Fig 4A). Tb.Th. of newly formed bone in the regenerate was significantly higher following the application of larger amplitude movements (Group 0.1: 0.06 mm; SD ± 0.02, Group 0.6: 0.1 mm; SD ± 0.02; *p* = 0.006; Fig 4B). Furthermore, Tb.N. in Group 0.6 (1.82 mm^-1;^ SD ± 0.94) was more than double that of Group 0.1 (0.78 mm^-1;^ SD ± 0.34), albeit not significant after post hoc correction (Fig 4C). Tb.Sp. was the only bone structural parameter not showing major differences between groups (Group 0.1: 0.42 mm, Group 0.6: 0.31 mm; Fig 4D).

### Histology

**Fig 5:**
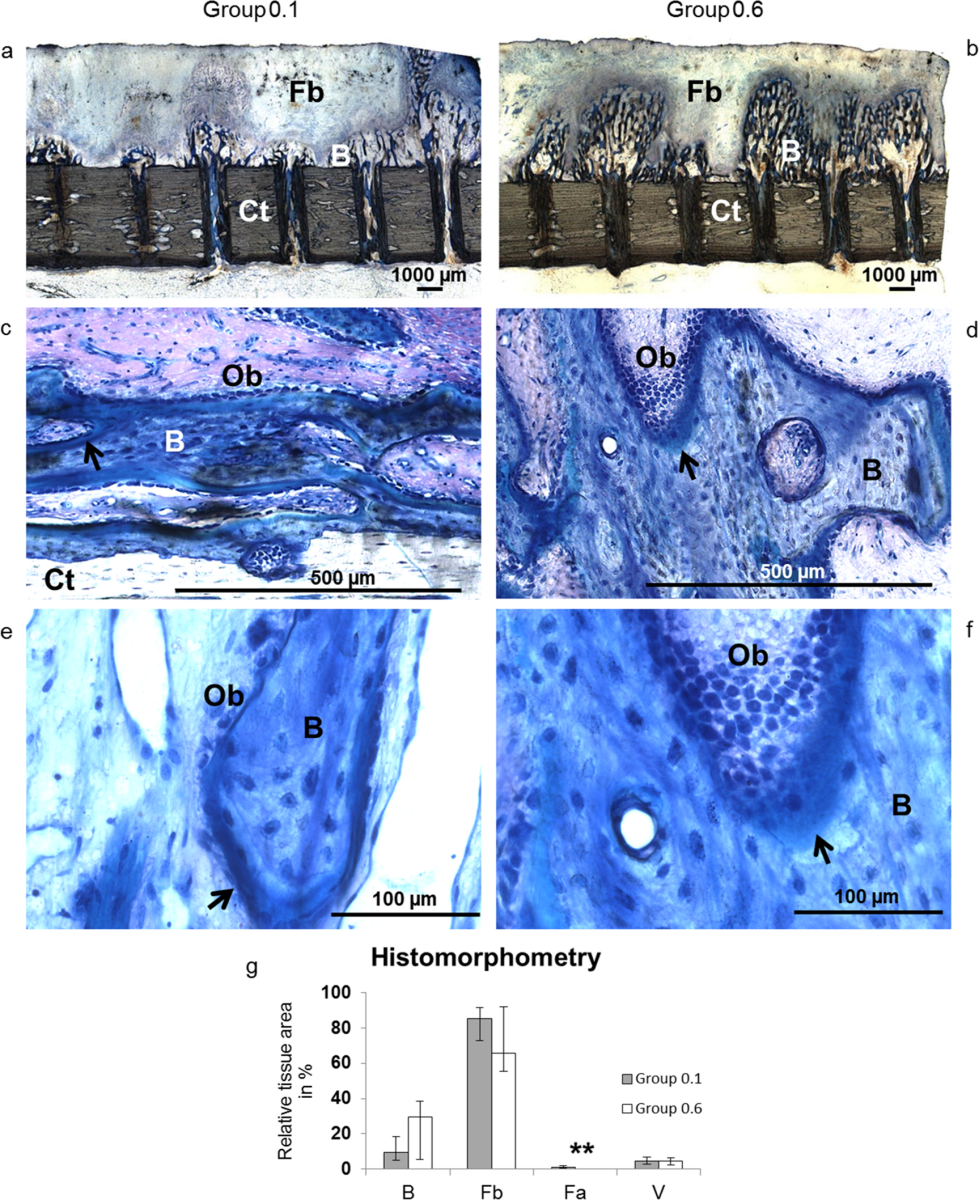
**a-g. Undecalcified histology and histomorphometry.** The representative histological images of Group 0.1 (left) and Group 0.6 (right) show spicules of newly formed bone (B) ascend from the cortex (Ct) into the regenerate gap in line of the medullary bore holes (Fig 5A and 5B). A faint layer of mesenchymal condensations covers the spicules and converts to fibrous tissue (Fb). There is no indication of endochondral ossification. At higher magnifications, animals from Group 0.1 (Fig 5C and 5E) and Group 0.6 (Fig 5D and 5F) show osteoblasts (Ob) lining layers of osteoid (arrows) around trabeculae of newly formed bone (B). 12.5-fold (a-b), 200-fold (c-d) and 400-fold (e-f) magnification. Histomorphometry (Fig 5G) shows differences of bone (B), fibrous tissue (Fb), fat cells (Fa, **p = 0.008) and vessels (V) between groups (grey bar: Group 0.1, white bar: Group 0.6; data given as median and range).

Histology showed no qualitative differences regarding tissue and cell differentiation. The regenerate was composed of woven bone in progressive maturity states that had grown from the base of the transcortical boreholes towards the titanium plate (Fig 5A and 5B). There was a constant layer of organized collagen fiber bundles and fibroblast-like cells covering the spicules reminiscent of mesenchymal condensations. The remainder of the gap was filled with vascularized fibrous tissue. Ossification was purely intramembranous. Safranin O staining did not indicate any signs of proteoglycans. There were neither morphological signs of terminally differentiated chondrocytes nor endochondral ossification. Osteoblasts were highly active with intensely stained, inflated nuclei representing increased metabolism (Fig 5C–5F). Osteoblasts occurred in multiple layers and exhibited extensive cellular polymorphism in Group 0.6.

Histomorphometric analysis revealed relative bone content comparable with μCT data (Group 0.1: 9.5%, range: 4.8–18.5; Group 0.6: 29.4%, range: 5.5–38.5). Fibrous tissue composed 85.1% (range: 72.8–91.5) in Group 0.1 and 65.8% (range: 55.3–92.0) in Group 0.6. Relative vessel volume showed no differences between experimental groups (Group 0.1: 4.5%, range: 2.7–6.7 and Group 0.6: 4.8%, range: 2.5–6.3). There was a significantly higher percentage of fat cells in Group 0.1 albeit on a low scale (Group 0.1: 1.2%, range: 0.5–2.0; Group 0.6: 0%, range: 0.0–0.1; *p* = 0.008) (Fig 5G).

### Immunohistochemistry

**Fig 6:**
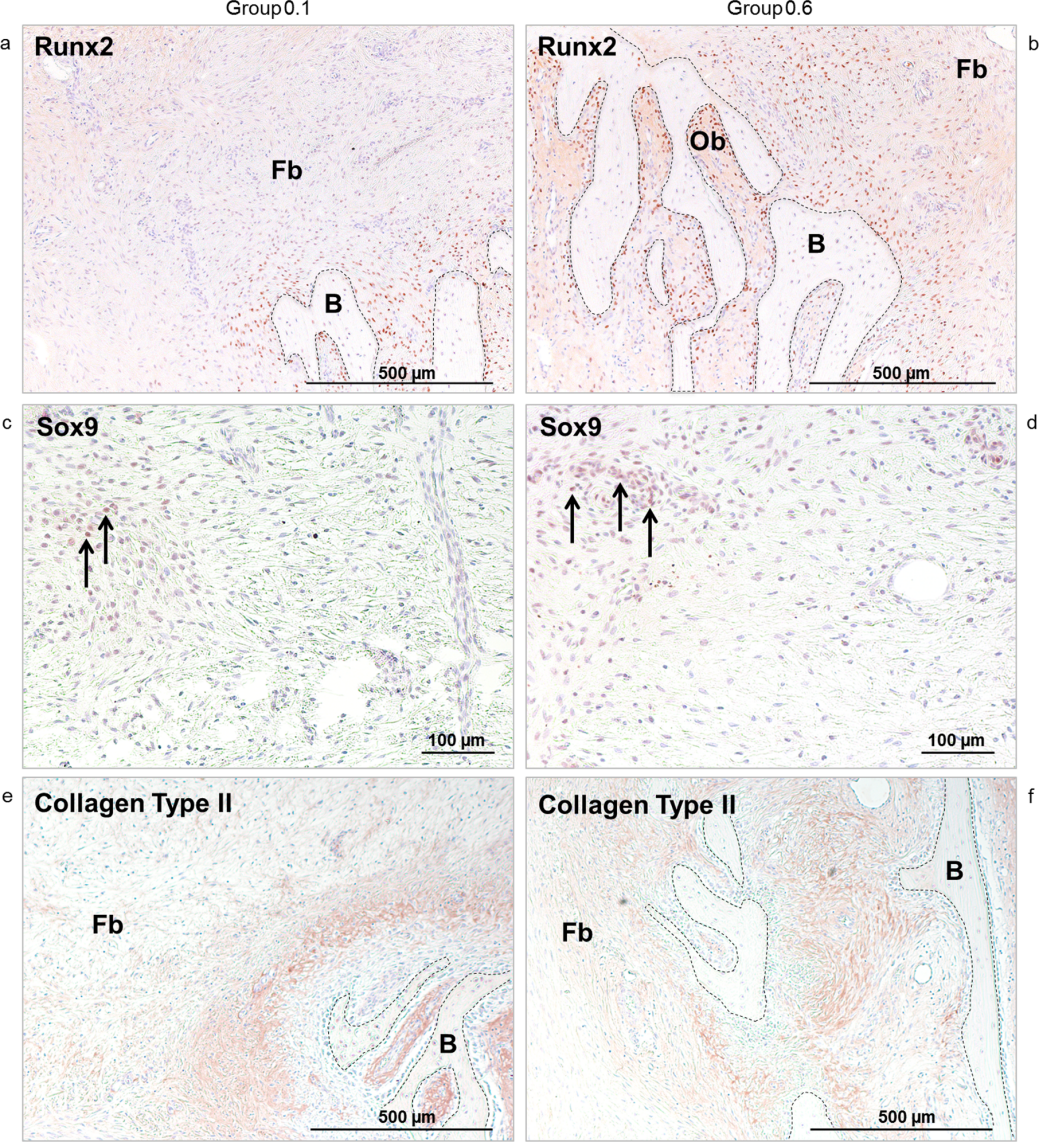
**a-f. Immunohistochemistry.** The intensive red to brown coloring indicates osteoblasts (Ob) and fibroblast-like cells staining positive for Runx2 (Fig 6A and 6B). Osteo-chondroprogenitor cells in the zone adjacent to the titanium plate surface are positive for Sox9 (arrows) (Fig 6C and 6d). Collagen Type II positive areas are visible as red to brown coloring in the collagenous matrix surrounding newly formed bone (B) but not in the fibrous tissue (Fb) distant to it (Fig 6E and 6F). Pictures on the left represent Group 0.1, pictures on the right represent Group 0.6. 50-fold (a-b, e-f) and 100-fold (c-d) magnification.

Expression of Runx2 was found in cells surrounding trabecular structures of bone forming spicules in the layer of mesenchymal condensations and underneath the titanium plate surface. The most intensely stained cells were osteoblasts adjacent to trabeculae, followed by fibroblast-like cells in close proximity to the spicules (Fig 6A and 6B). A few osteocytes exhibited a weak positivity. The number of cells staining for Runx2 correlated with the amount of new bone, even in areas further from the cortical surface, and appeared higher in Group 0.6. Sox9 positive cells were found in a layer close to the titanium plate surface in both experimental groups (Fig 6C and 6D). Devoid of a notable distribution pattern, a few cells neighboring osseous structures also showed a weak positivity. Collagen Type II positive matrix was seen adjacent to trabeculae within bone spicules and at their periphery. In Group 0.6, positive areas exhibited a wider distribution (Fig 6E and 6F). Occasionally, Collagen Type II positive vesicles were observed intracellularly.

## Discussion

In this study, the effects of cyclic purely axial compression amplitudes on bone formation after callus distraction were investigated. The results showed that the larger compression amplitude (Group 0.6 mm) representing moderate compressive interfragmentary movements (IFM) stimulated significantly more new bone formation than the small compression amplitude (Group 0.1 mm), but in both groups only intramembranous bone formation was observed.

The finding that moderate predominantly compressive amplitudes stimulate more bone formation than small compression amplitudes or rigid fixation is in agreement with previously published osteotomy callus distraction models [4–6]. In sheep metatarsi, axial compression of 0.5 mm led to greater bone formation than compression with 0.18 mm [6]. Similar results are known for fracture healing studies in which greater predominantly compressive IFM led to higher callus density [13, 14]. Moderate compression at 10% strain increased osteogenic activity of bone cells [15]. In contrast to the experiment described herein, endochondral ossification and cartilage formation could be detected in previous callus distraction and fracture healing studies submitted to moderate predominantly compressive stimulation [6, 13, 16]. Although Sox9 positive cells and Collagen Type II could be detected in the current experiment, there were no terminally differentiated chondrocytes or proteoglycan production. Because Runx2 and Sox9 were co-expressed in the same region, Sox9-positve cells were suggested to be osteo-chondroprogenitors as previously described [17].

There are three possible explanations for the pure intramembranous bone formation seen in this experiment as well as for the endochondral bone formation additionally seen in other studies focused on the influence of cyclic compression: differences in direction of IFM, the vascularization in the healing zone, and a possible triggering of osteogenic cell differentiation by tensile strain during distraction may all account for the discrepancies.

Firstly, stimulation of the regenerate tissue after the callus distraction process was solely by controlled cyclic compression in the present model. Tissue deformation due to movements in other directions was avoided by the stiff fixator-system connected to the intact tibia. In osteotomy models previously described [1, 6, 16, 18], other movements, such as bending and shear, could not be completely prevented. Cyclic external loads and muscle forces act within the osteotomy site and the bridging fixator systems cannot be perfectly rigid. Therefore, unquantifiable, auxiliary IFM is present alongside the mechanical motion parameters of interest in osteotomy models. An experiment on different hybrid external fixators reported that shear movement occurs *in vitro* even under pure axial compression [19] and shear movement generally exceeded axial compression in patients with correction osteotomies [20]. From fracture healing studies it is well known that shear movements are particularly detrimental for the healing process and induce more cartilage formation [21–23]. Low fixator stiffness was identified as one cause of endochondral ossification, and the more oblique tibia loading delayed healing compared to axially loaded radii in DO studies on dogs [1, 24]. López-Pliego et al. [18] have indicated that endochondral ossification during distraction osteogenesis has been reported primarily in small animal studies while intramembranous ossification is dominant in large animal models. They explain this result as a function of fixator stiffness as small animal models typically employ flexible monolateral fixators. In contrast, large animal models allow the technical freedom to develop stiffer and more controllable fixation devices. In the study of López-Pliego et al., 10 adult, female, Merino sheep (the same as those used in the present work) were subjected to bone segment transfer using a stiff fixator and transport protocol of 1 mm per day, once daily for 15 days. Subsequently, the specimens were euthanized at 10 different time points between 2 and 510 days after the completion of distraction. Despite the much larger rate of distraction in comparison to that of the present work the, the authors report that foci of endochondral ossification could only be seen in only 3 of the 10 specimens. When considering that the model of López-Pliego et al. was subject to the imperfect stability associated with osteotomy, it seems likely that the appearance of endochonodral ossification in some specimens was due to variation in interfragmentary motion, exceeding some critical threshold in those specimens. Such variations in interfragmentary motion can easily result from surgical variation, interindividual loading conditions among specimens, as well as manufacturing variation of mating parts for each fixator employed. The model described in the present work avoids such issues by eliminating the osteotomy and therefore interfragmentary motion associated with loading and fixation stiffness. Therefore the prevention of shear movement in the present study may be one of the possible reasons for pure intramembranous bone formation.

A second reason for the appearance of endochondral ossification and cartilage formation in osteotomy models of DO [1, 6, 16] but not in our experiment might be the difference in vascularization of the regenerating zone. Blood supply is a prerequisite for intramembranous bone formation whereas endochondral ossification can take place under hypoxic conditions [25, 26]. Drilling bore holes into the cortical wall of the tibia allowed neovascularization of the regenerating tissue by vessels penetrating from the medullary cavity. This led to a very good vessel density as detected in our histological evaluation. The larger compression amplitude during maturation did not affect vessel density which was on par with values from the animals of the low amplitude compression group. Omitting drill holes in the current model, however, significantly suppressed bone formation [7]. In callus distraction models, differences in blood supply were found when full osteotomy models were compared to more vascularity-preserving techniques such as corticotomies [1]. After corticotomy in dogs, the distraction gap bridged by intramembranous ossification even under fixation exhibiting insufficient stability [27]. Therefore, chondrogenic differentiation may occur in either primarily hypoxic sites and/or under shear movement hindering neovascularization. Frierson et al. associated fibrocartilage development after DO with either wire loosening which likely induces shear movement, or the absence of neovascularization [28].

A third reason may be that the distraction process stimulates pure osseous differentiation in regenerating tissue and this influence persists during the maturation phase. Runx2 up-regulation was found in biopsies of tissue regenerates in human patients [29]. and pure intramembranous bone formation was seen in sheep with solitary lateral callus distraction avoiding IFM during the maturation phase in the predicate experiment [7]. Mesenchymal stem cells show up-regulation of osseous genes under tensile strain whereas compression leads to expression of genes representing cartilaginous differentiation [30]. The strain stimulus for osseous differentiation does not stop once the distraction process has finished but rather persists for a significant period of time. The process of calcification of the young regenerate takes about twice as long as the distraction period [1]. Even without any further mechanical stimuli, intramembranous bone formation persists for at least 50 days after callus distraction [7]. The “Tension-stress” effect may superimpose with the stimulus induced by cyclic compression applied in the current experiment. Thus, osseous differentiation may have suppressed or restricted the stimulus for the differentiation of cells to chondrocytes through cyclic compression [3]. Previously, this could be demonstrated when a limited number of strain stimuli where applied in an eight week fracture healing study stabilized by an external fixator [31]. Eight days of small distractions (two-times 0.5 mm/day) followed by a subsequent shortening to the original size of osteotomy gap led to an improved healing process in comparison to a non-stimulated control group. Although the number of IFM cycles due to weight bearing of the sheep and the elasticity of the fixator system far outweighed the few tensile strain stimulations, there was a pronounced osteogenic effect of the tensile stimuli. More bone formation and less cartilage tissue was observed in the stimulated group in comparison to the control group with constant flexible fixation of the fracture. These studies show that tensile strain applied during the distraction phase, triggers cell differentiation in the osseous direction and can partially suppress differentiation to chondrocytes under moderate cyclic compression. This is supported by the result of this study which shows intensive Runx2 staining of cells in the group with the larger compression amplitude (0.6 mm) and no sign of terminal cartilaginous differentiation.

It could be discussed whether the resection of the periosteum in the callus distraction area reduces the capability of precursor cells to differentiate to chondrocytes because the periosteum is a major source of chondrocytes [32]. However, analysis of bone formation directly next to the callus distraction area (outside the area of interest) where the periosteum was kept intact also showed only intramembranous bone formation. This indicates that the main source of precursor cells and osteoblasts in the callus healing area are coming from the vessels originating from the bone marrow and that the “tension-stress effect” may have superimposed with the possible effect of cartilage formation induced by cyclic compression.

Whether much larger cyclic compression strains will hinder pure intramembranous bone formation needs further investigations.

### Conclusion

Pure tensile strain in the callus tissue during the distraction procedure induces a strong osteogenic differentiation of mesenchymal precursor cells. This stimulus persists beyond the distraction phase. The mode of intramembranous bone formation remains unaffected when pure cyclic compression is applied after distraction. In this study, the larger amplitude of cyclic compression led to an increased intramembranous bone formation compared to the small amplitude. So far, this effect has only been demonstrated during endochondral ossification. Whether there is a critical value of compressive strain that hinders intramembranous bone formation cannot be definitively answered and needs further investigation.

From a clinical perspective, callus distraction should be performed using a fixation system which eliminates as much shear movement perpendicular to the bone axis as possible following a vascularity-preserving surgical technique. With regards to axial movements, more IFM than expected is tolerable and stimulating for intramembranous bone formation.
